# A Quantitative Risk Assessment Model Involving Frequency and Threat Degree under Line-of-Business Services for Infrastructure of Emerging Sensor Networks

**DOI:** 10.3390/s17030642

**Published:** 2017-03-21

**Authors:** Xu Jing, Hanwen Hu, Huijun Yang, Man Ho Au, Shuqin Li, Naixue Xiong, Muhammad Imran, Athanasios V. Vasilakos

**Affiliations:** 1 College of Information Engineering, Northwest A & F University, Yangling 712100, China; jingxu@nwsuaf.edu.cn (X.J.); Heaven_hu@yeah.net (H.H.); yhj740225@nwsuaf.edu.cn (H.Y.); 2 Department of Computing, The Hong Kong Polytechnic University, Hong Kong 999077, China; csallen@comp.polyu.edu.hk; 3 Department of Mathematics and Computer Science, Northeastern State University, Tahlequah, OK 74464, USA; 4 College of Computer and Information Sciences, Almuzahmiyah, King Saud University, Riyadh 11543, Saudi Arabia; cimran@ksu.edu.sa; 5 Department of Computer Science, Electrical and Space Engineering, Lulea University of Technology, Lulea 97187, Sweden; th.vasilakos@gmail.com

**Keywords:** cloud computing, line-of-business services, access control, risk assessment, intrusion effort

## Abstract

The prospect of Line-of-Business Services (LoBSs) for infrastructure of Emerging Sensor Networks (ESNs) is exciting. Access control remains a top challenge in this scenario as the service provider’s server contains a lot of valuable resources. LoBSs’ users are very diverse as they may come from a wide range of locations with vastly different characteristics. Cost of joining could be low and in many cases, intruders are eligible users conducting malicious actions. As a result, user access should be adjusted dynamically. Assessing LoBSs’ risk dynamically based on both frequency and threat degree of malicious operations is therefore necessary. In this paper, we proposed a Quantitative Risk Assessment Model (QRAM) involving frequency and threat degree based on value at risk. To quantify the threat degree as an elementary intrusion effort, we amend the influence coefficient of risk indexes in the network security situation assessment model. To quantify threat frequency as intrusion trace effort, we make use of multiple behavior information fusion. Under the influence of intrusion trace, we adapt the historical simulation method of value at risk to dynamically access LoBSs’ risk. Simulation based on existing data is used to select appropriate parameters for QRAM. Our simulation results show that the duration influence on elementary intrusion effort is reasonable when the normalized parameter is 1000. Likewise, the time window of intrusion trace and the weight between objective risk and subjective risk can be set to 10 s and 0.5, respectively. While our focus is to develop QRAM for assessing the risk of LoBSs for infrastructure of ESNs dynamically involving frequency and threat degree, we believe it is also appropriate for other scenarios in cloud computing.

## 1. Introduction

As one significant recent advance in the field of information technology [1], cloud computing [2] provides access to a shared pool of configurable computing resources (e.g., services, applications, networks, storage and servers) that is convenient for management and service provider interaction. It can be classified according to the service models, such as Software as a Service (SaaS), Platform as a Service (PaaS), and Infrastructure as a Service (IaaS) [2]. SaaS can be characterized as: software deployed as a hosted service and accessed over the Internet [3]. As a completely innovative hosted application service model, SaaS is one of development directions of software delivery. It has become an important way for small and medium enterprises to acquire advanced technology. According to the service type provided by the Service Provider (SP), SaaS can be further divided into two major categories, namely: consumer-oriented services and Line-of-Business Services (LoBSs) [3]. Offering to enterprises and organizations of all sizes, LoBSs are often large, customizable business solutions aimed at facilitating business processes, such as finance, supply-chain management, and customer relations. They are typically sold to customers on a subscription-basis [3].

Emerging Sensor Networks (ESNs) increase the requirements of computing and communication technologies and systems, which should integrate technologies and methods with respect to resource allocation, data mining, knowledge sensing, and intelligent control in order to satisfy the variety of applications in industry and business [4]. ESNs are an inevitable trend with the development of the Internet of Things (IoT), and intend to connect almost every intelligent device [4]. The infrastructure of LoBSs may be ESNs in different application domains, including, for example, the medical equipment of digital cameras, smart phones and medical imaging equipment [5], and vehicle detection in autonomous vehicles, driver assistance systems, intelligent parking systems, or measurement of traffic parameters [6], and monitoring and assessing fruit freshness in Internet-of-Things-based e-commerce delivery using scenario analysis and interval number approaches [7], and helicopters and vehicles’ intermodal transportation of medical supplies [8]. In LoBSs, SP provides Internet-based software delivery and services to the tenants in which the common software features sink into the infrastructure and its operation and maintenance are provided by SP. At the same time, the tenants (business or organization) consume and utilize the services as a user but not the owner [9]. This paper focuses on LoBSs for infrastructure of ESNs, for its numerous advantages such as on-demand self-service, extensive network access, resource sharing, rapid scalability, scalable services, and increasing the quantity of tenant and information by geometric curve.

The prospect of LoBSs is exciting, but the security problem has become one of main obstacles hindering its development. Many ways to protect LoBSs have been investiaged, such as fairly sharing the sensitive information between two tenants [10,11,12], and the messages embedded by spatial least significant bit [13], and arithmetic privacy homomorphism [9]. However, they cannot fully balance LoBSs’ contradictory requirement between flexible access and absolute security. As both business’s application and database are deployed on SP’s server, the ownership of both business’s application and database are separated from its usufruct. The same is true for the ownership and maintenance of data [9]. Thus, access permission is a top challenge in LoBSs’ security.

In contrast to a conventional attacker that needs to undertake huge efforts to break through the access control, a LoBSs’ attacker only need to register as a legitimate user to gain access to the system. In other words, most intruders in LoBSs are simply eligible users that misbehave. Since users who have legitimate access are the cause of the security risk, authenticating each user once only during a session cannot meet the security demand. A large number of valuable resources are gathered in SPs, which has great appeal to attackers. To illegally obtain resources, an attacker may break through the restrictions of access control by various means. In the traditional network information system, there is a trusted domain including data, user, server, and so on, where the data is stored on the trusted server maintained by the trusty user, and the user’s eligibility to access data is authenticated by the server, and the users are constrained by the entity organization in addition to remaining within the bounds in the virtual system. In LoBSs, there is not a trustworthy domain as the users are only kept within the virtual system. By operating as a legitimate user, an attacker could attack a SP’s servers to expose the privacy and sensitive data of subscriber users [5]. More concretely, any attack will appear as a series of malicious acts. In order to protecting a SP’s server, and ensure an acceptable quality of services in a relatively unpredictable network environment [14] to subscribers, LoBSs’ risk must be assessed dynamically. With the growing popularity of network applications, the potential security risks increase sharply. To protect an information system, it is of prime importance to assess the information security risks [15]. Risk is a likelihood function in which a particular potential vulnerability may be exercised by a given threat-source and the adverse event maybe impact on the organization [16]. To avoid the loss of resources, after the information system risk is assessed dynamically, the access permission should be adjusted dynamically. Jing et al. [17] researched the risk of cloud servers from malicious users dynamically to adjust users’ access rights based on the risk of the user’s operation. It does not, however, consider the effect of frequency and threat degree of malicious operations. The higher is the frequency of a malicious operation, the higher is the LoBSs’ risk. The higher is the degree of threat of a malicious operation, the higher is the LoBSs’ risk. In order to quantify LoBSs’ risk more precisely, the impact both frequency and threat degree of user behavior must be involved. 

Motivated by the above observation, based on Value at Risk (VaR) [18], we propose herein the Quantitative Risk Assessment Model (QRAM) which takes frequency and threat degree into consideration to assess LoBSs’ risk. The model consists of the following components: first, the threat degrees of malicious act are graded based on the Snort user manual [19] and quantified in equidistant division. Second, the elementary intrusion effort is quantified based on a network security situation assessment model [20]. Third, the intrusion trace effort is quantified based on multiple behavior information fusion [21]. Fourth, LoBSs’ objective risk is quantified based on the rate of weighted threats in intrusion traces. Fifth, LoBSs’ subjective risk is quantified based on the Shannon entropy [22] of experts’ scores. Sixth, LoBSs’ comprehensive risk is quantified on both the intrusion trace probability and the proportion between subjective risk and objective risk. Seventh, under the influence of intrusion traces, QRAM involving frequency and threat degree is proposed to dynamically assess LoBSs’ risk by the historical simulation method of VaR [18]. Besides LoBSs, we note that QRAM can be applied to cloud computing in general, even prompted to the application layer multicast [23]. The special contributions of this paper include:

- The efforts of both elementary intrusion and intrusion trace are quantified based on the evaluation for security situation of networked systems.
- The subjective risk is determined based on Shannon entropy of experts’ scoring.
- QRAM involving frequency and threat degree is proposed to quantify LoBSs’ risk based on VaR.

The rest of this paper is organized as follows: Section 2 discusses related works, followed by preliminaries in Section 3. The intrusion effort involving frequency and threat degree is assessed in Section 4. The quantitative risk assessment model involving frequency and threat degree is proposed in Section 5. Section 6 shows the simulation test and discussion, and Section 7 concludes the paper with a summary and some future research directions.

## 2. Related Works

We review some related works including user behavior analysis and prediction, user behavior analysis and trust management, user behavior risk assessment and trust management in this section.

### 2.1. User Behavior Analysis and Prediction

Based on the analysis of prevalent network intrusions about multiple behavior information fusion, a new model of security threat evaluation [21] was presented with a set of quantitative indexes. In order to defend against application layer distributed denial-of-service attacks, an anomaly detection based on web user access behavior [24] was proposed, in which the web user’s browsing behavior is observed at a web server by a hidden semi-Markov model. Concerning cloud computing, Tian et al. [25] mainly researched the evaluation importance of evaluation strategy and user behavior trust, including the basic idea of evaluating user behavior trust, principles for evaluating user behavior trust, evaluation strategies of behavior trust for each access, trust object analysis and long access. In terms of incomplete information multi-stage dynamic games, Chen et al. [26] proposed a behavior analysis model, where both negative and positive false factors are considered in network detection methods, and the actions both current and historical improved the comprehensiveness and accuracy of the dynamic judgment for end-use trustworthiness. Chen et al. [27] investigated the characteristics of cloud computing requests received by the cloud infrastructure operators. These cluster usage datasets, released by Google, were thoroughly studied. These researches could address the self-similarity and non-stationary characteristics of the workload profile in a cloud computing system. Ashwini et al. [28] discussed user browsing behavior and interest, and web mining technology, web log data.

### 2.2. User Behavior Analysis and Trust Management

A trust quantification algorithm [29] was presented based on grey fuzzy theory, and a new trust-based dynamic access control model [29] was proposed, which used the arcsine function to construct an algorithm to perform mapping between trust values and access permissions for effective access control. With the trust levels’ idea for identity management, Parikshit et al. [30] proposed a fuzzy approach to the trust based access control, which was sealed with the linguistic information of devices to descript access control in the Internet of Things. Jaiganesh et al. [31] proposed a fuzzy logic technique called fuzzy ART, where the consumption of resources was periodically scanned, and the virtual machine states were classified into categories from stable to attackers based on the traced-out behaviors. Kambiz and Mehdi et al. [32] used not only the trust manager component, but also machine learning for the system to learn from the user’s behavior and recognize access patterns, which not only limited the illegitimate access, but also predicted and prevented potential malicious events and questionable accesses.

### 2.3. User Behavior Risk Assessment and Trust Management

Zhang et al. [33] proposed a trust model based on behavior risk evaluation, which established a set of feature matching rules based on asset identification, vulnerability identification and threat identification for the system, constructed a complex weighting function to compute the potential risk implied in behaviors of the entities, and designed a trust computation method based on risk. Xu and Dou [34] proposed a risk evaluation model based on asset evaluation, vulnerability evaluation and threat evaluation by identifying and quantifying the risk factors, in which the value, vulnerability and threat of asset were combined to compute the system risk, and a risk computation method merging behaviors trust of external entities was presented using the quantitative calculation of information entropy weight of each factor for overcoming subjectivity of direct assignment considering the risk of system was influenced by the behavior of external entity. Jing et al. [14] proposed the user behavior assessment based dynamic access control model by introducing user behavior risk value, user trust degree and other factors into role based access control.

Three aspects of user behavior in network systems is reviewed: user behavior analysis and prediction, user behavior analysis and trust management, user behavior risk assessment and trust management. Under different conditions, user behavior has different characteristics. Frequency and threat degree of user behavior in cloud services is not involved, nothing is retrieved about user behavior under LoBSs.

## 3. Preliminaries

In this section, we introduce the notations and related technologies deployed in our scheme.

### 3.1. Definitions

**Definition** **1.** *Elementary intrusion: any alert that is evoked by intrusion behavior and reported by an intrusion detection system, described by A [aid] = {src, dst, sp, dp, t, type, sensor, sig}, whose elements’ descriptions are as listed in Table 1* [21].

**Definition** **2.** *Intrusion trace: a trace constituted of a series of elementary intrusions that user privilege has allowed to occur illegally, defined as C[cid] = {{src_1_, dst_1_, dp_1_, seq_1_, aid_1_, count_1_, sig_1_}, {src_2_, dst_2_, dp_2_, seq_2_, aid_2_, count_2_, sig_2_},…}* [21].

**Definition** **3.** *Intrusion effort: effort expend by an individual hacker to get user privileges along an intrusion trace* [21].

**Definition** **4.** *Mean intrusion effort: according to the difficulty’s degree of each elementary intrusion in the intrusion trace, the difficulty’s degree to get user privileges for the target is measured* [21].

**Definition** **5.** Time window frequency: the ratio between the number of a given level intrusions happening and the total number of elementary intrusions happened in a unit of time.

**Definition** **6.** Rate of weighted threat in intrusion trace: the summation of the products of the severity of the malicious behavior at different levels by the time window frequencies in units of time.

**Definition** **7.** *Confidence interval: an observed interval, in theory distinct from sample to sample, that often includes the value of an unobservable parameter of interest if the experiment is duplicated* [35].

**Definition** **8.** *Confidence level: the proportion of such intervals that include the true value of the parameter will adapt the given confidence level if the confidence intervals are structured by a lot of separate data analyses of replicated (and possibly different) experiments* [35,36,37].

### 3.2. Shannon Entropy

Shannon entropy [22] is the expected value (average) of the information contained in each message. Supposed the entropy *Η*(*X*) [38] of a discrete random variable *X* with possible *n* values {*x*_1_, …, *x_n_*} and probability mass function *Pr*(*X*) is defined as:

(1) $$H(X)=\sum_{i=1}^{n}Pr(x_{i})I(x_{i})=-\sum_{i=1}^{n}Pr(x_{i})\log_{b}Pr(x_{i})$$

where *I* is the information content of *X* [39,40], *I*(*X*) is itself a random variable, and *b* is the base of logarithm used. Common values of *b* are 2, Euler’s number *e*, and 10, and the unit of entropy is Shannon for *b* = 2, nat for *b* = *e*, and hartley (unit) for *b* = 10 [37]. When *b* = 2, the units of entropy are also commonly referred to as bits. Shannon entropy is characterized by a small number of criteria, any definition of entropy satisfying the assumption as the form [41]:

(2) $$-K\sum_{i=1}^{n}p_{i}\log(p_{i})$$

where *K* is a constant corresponding to a choice of measurement units, and *p_i_* = *Pr*(*X* = *x_i_*) is the probability of *x_i_*, and *Pr*(*x_i_*) is the probability function of *x_i_*.

### 3.3. Historical Simulation Method of Value at Risk

As a measure of investments’ risk, VaR assesses how much a group of investments might lose [42]. Supposed a confidence level (Definition 8) *α* ∈ (0, 1), the VaR of portfolio at the confidence level *α* is given by the smallest number *l* such that the probability *Pr*(*L* > *l*) that the loss *L* exceeds *l* is at most (1 − *α*) [43]. Mathematically, if *L* is the loss of portfolio, then *VaR*_α_(*L*) [44] is the level *α*-quartile as:

(3) $$VaR_{\alpha }(L)=\inf\{l\in R:\mathrm{Pr}(L>l)\leq 1-\alpha \}=\inf\{l\in R:F_{L}\geq \alpha \}$$

The keys to calculate VaR include the speculation of future changes in market factors and the relationship between portfolio value and market factors (linearity, non-linearity). The fundamental computation methods of speculation of future changes in market factors include historical simulation method, parametric method and Monte Carlo method [45].

As a nonparametric method, the core of historical simulation methods is to simulate the future income distribution of portfolio based on historical sample changes, and then use the quartile to calculate the VaR estimation under certain confidence. The method calculates the full value of portfolio rather than the local approximation of a small change in price. At the same time, this method avoids the simulation risk by using real data, and does not need to make specific assumptions on the distribution, nor need to estimate the parameters, so it can deal with the asymmetric and rear tail problems. Because the historical data reflect the simultaneous changes of all risk factors in the market, the problems of volatility, correlation and back-end issues can be reflected in real historical data, which often need to be considered separately.

The general method makes on assumption about the shape of the distribution of returns. Define *W*_0_ as the initial investment and *R* as its rate of return, which is random. Assuming that the position is fixed, or that there is no trading, the portfolio value at the end of the target horizon is *W* = *W*_0_ (1 + *R*) [46]. The expected return and volatility of *R* are defined as μ and σ. Define now the lowest portfolio value at the given confidence level *c* as W^*^ = *W*_0_ (1 + R^*^) [46]. VaR measures the worst loss at some confidence level, so it is expressed as a positive number. The *relative VaR*(*mean*) [46] is defined the dollar loss relative to the mean on the horizon as:

*VaR* (*mean*) = *E* (*W*) − W^*^ = −*W*_0_ (R^*^ − *μ*) (4)

Often trading VaR is defined the *absolute VaR* [46], that is, the dollar loss relative to *zero* or without reference to the expected value as:

*VaR* (*zero*) = *W*_0_ − W^*^ = −*W*_0_R^*^ (5)

At a given confidence level *c*, we wish to find the worst possible realization W^*^ such that the probability of exceeding this value is *c* [46], that is:

(6) $$c=\int_{W^{*}}^{\infty }f(w)dw$$

The probability of a value lower than W^*^, $p=Pr(w\leq W^{*})$, is 1 − *c* [46], that is:

(7) $$1-c=\int_{-\infty }^{W^{*}}f(w)dw=Pr(w\leq W^{*})=p$$

The number W^*^ is called the quartile of distribution, which is the cutoff value with a fixed probability of being exceeded. Note that we did not use the standard deviation to find the VaR. The historical simulation methods have the above advantages, but there are still some limitations. One is that they are entirely dependent on specific historical data, that is, it is assumed that the future situation and the performance of historical data in the past will be the same, but in fact, some of the past impact of the loss of events in the future does not necessarily repeat itself, and future events may also never have occurred in the past. The other is that they are likely to be limited by the amount of data, not fully reflect the risk of all situations, such as some extremes unlikely to happen.

## 4. The Intrusion Effort Involving Frequency and Threat Degree

Before assessing LoBSs’ risk, the intrusion efforts of both elementary intrusions and intrusion traces should be calculated. The elementary intrusion effort under threat degree is quantified based on network security situation assessment model. The intrusion trace effort under frequency effects is quantified based on fusion of multiple behavior information.

### 4.1. The Overall Framework to Assess the Intrusion Effort

To study the impact of malicious operation on LoBSs’ risk, the intensity of attack is described by the intrusion effort, which includes both the element intrusion and the intrusion trace. Situational awareness [47] is the ability to evaluate, process, and understand the information of critical elements about what is happening to the team regarding the mission. The security situation assessment [48] is an effective means to quantify network security, which refers to perceiving and obtaining the security-related elements through technical means from time and space dimensions, to determine the security situation through integrated analysis of data and to forecast its future trends. Aiming at the deficiency that is unable to provide useful security information encountered in the current security evaluation systems, the log database of intrusion detection system is led to the hierarchical and quantitative model, which is used to evaluate the security situation of network system, and its corresponding computation method are proposed based on the importance of service, host, and the structure of network system [20].

**Figure 1:**
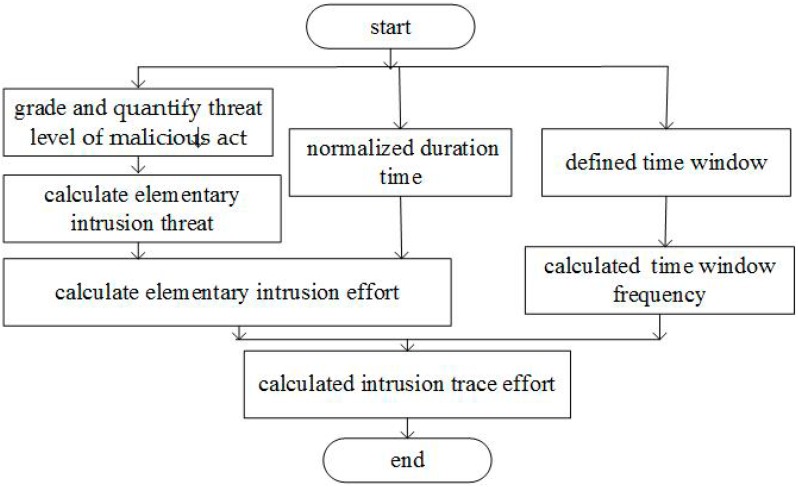
Calculation process of intrusion effort.

Based on the thought of everything as a service [49], the resources of hardware, software and data are provided as a service, so user behavior of LoBSs belongs to a service. Base on the hierarchical and quantitative model [20], the calculation process of intrusion effort involving frequency and threat degree is proposed as Figure 1.

Step 1 The threat level of malicious act is graded and quantified by the threat index.
Step 2 Amending the network security situation assessment model, the elementary intrusion threat is calculated.
Step 3 By combining with the duration which is normalized, the elementary intrusion effort is calculated.
Step 4 By combining the time window frequency, the intrusion trace effort is calculated.

### 4.2. Threat Degree of Elementary Intrusion

LoBSs have the characteristics of openness and sharing [3], so the attacks against SPs’ servers are becoming more and more common. Any attack is achieved through a series of malicious behaviors, which must pose a risk to the SP’s server. The threat degree to LoBSs varies depending on the severity degree of attacks. In order to quantify LoBSs’ risk, the attacks should be graded by their harmful level. These attack classifications are listed in Table 2 [19].

It can be seen from Table 2 that the attacks are currently graded with four default priorities, such as very low, low, medium, and high, in which a priority of 4 (very low) is the least severe, and 1 (high) is the most severe. Based on the severity degree of attacks from low to high, the most common attacks listed are host discovery, port scanning, privilege escalation, denial of service, and covert scanning [20]. The Snort attack classifications are divided in equidistant divisions. In the equidistant division of attacks, the priorities of attacks from low to high are quantified such as 0.2, 0.4, 0.6, 0.8 and 1 as shown in Table 3.

Assuming that an undefined degree of harm behavior is a threat with a very low level, its initial quantization level is 0.2. While quantifying LoBSs’ risk, the threat degree should be updated in time based on detected user behavior.

### 4.3. Elementary Intrusion Effort under Threat Degree

Because the occurrence of malicious acts is random and dynamic, and is independent of the past, it has the Markov property [50]. The Markov property of malicious acts leads LoBSs’ safety to change, so only the current state is involved in calculating the elementary intrusion effort. Since the current state corresponds to a time point, one cannot estimate the effect that the behavior at a time point has on LoBSs’ risk. In order to calculate the elementary intrusion effort, a very short period (such as 1 ms, 10 ms, etc.) is seen as a time point, that is, the fully malicious acts in the set period are treated as an elementary intrusion. Based on the theory of integrals, the elementary intrusion effort is assessed during the set period (such as 1 ms, 10 ms, etc.).

The elementary intrusion effort is related to factors such as threat degree, financial costs, duration, attacker’s experience, practicability of attack tools, attack time, counting ability, and so on [51]. Based on a principal component analysis [52], the factors of threat degree and frequency are mostly considered in this paper.

At the same time, a SP’s server may be attacked by different priority attacks from different sources. Suppose that the evaluation time is *t*, and the attack’s priority is i∈{4, 3, 2, 1, 0} which corresponds to {*high*, *medium*, *low*, *very low*, *unknown*} in Table 3, and the number of attacks of priority *i* during evaluation time *t* is *C_i_*, and the severity of the attacks is $W_{i}\in \{4,\;3,\;2,\;1,\;0\}$, in the network security situation assessment model, Hu et al. [53] proposed the network security situation *threat_i_* [53] under the attack severity *P_i_* as:

(8) $$threat_{i}=10^{W_{i}}$$

According to practical experience, the risk indexes for an event ocurring 100 times with severity 1, 10 times with severity 2 and 1 time with severity 3 [20] are equivalent, so the influence coefficient of the risk indexes is 10*^i^*, i∈{4, 3, 2, 1,0}. In order to accurately quantify the impact of threat degree on the risk index, the attack priority quantization is $P_{i}\in \{1,\;0.8,\;0.6,\;0.4,\;0.2\}$ in Table 3. Chen et al. [20] put forward the threat degree $C_{i}10^{P_{ij}}$ when optimizing *W_i_* in [53], which does not reflect the influence coefficient of risk indexes 10*^i^*, i∈{4, 3, 2, 1,0}, so it should be amended to $C_{i}10^{(i+1)P_{i}}$. Under the condition of $C_{i}10^{(i+1)P_{i}}$ and Equation (8), the network security situation *threat_i_*^’^ under a number *C_i_* of attacks of severity *P_i_* is optimized as:

(9) $$threat_{i}^{\prime }=10^{C_{i}10^{(i+1)P_{i}}},\;i\in \{4,\;3,\;2,\;1,\;0\}$$

Because a SP’s server in LoBSs may be attacked by different priority attacks from different sources at the same time, an elementary intrusion may comprise many attacks which are from different attackers at different threat levels. On the basis of Equation (9) and with reference to the mean intrusion effort approach [21], the threat degree *threat* of an elementary intrusion is improved as:

(10) $$threat=10^{\frac{\sum_{i=0}^{4}C_{i}10^{(i+1)P_{i}}}{100\sum_{i=0}^{4}C_{i}}},\;i\in \{4,\;3,\;2,\;1,\;0\}$$

The network security situation assessment model [21] focuses on a qualitative analysis, which uses a numerical range to illustrate the risk degree and the probability of occurrence of an attack. In Equation (10), multiple threats are combined using quantitative analysis rather than qualitative descriptions, so the calculated threat degree is more scientific and rigorous.

The dimensionless of attack duration *last* is defined as:

(11) last=lasttime10j

where *j* represents the ratio order in the duration affecting the factor of an attack, and which is set by experts.

As the main factors affect the intrusion effort, the threat degree is independent of the attack duration. When calculating their effect on the elementary intrusion effort, the addition principle in combinatorics is suitable. Integrating Equations (10) and (11), the elementary intrusion effort *ElemEffort* is proposed as:

*ElemEffort* = *threat* + *last* (12)

where *ElemEffort* represents the elementary intrusion effort, and *threat* represents the threat degree, and *last* represents the normalization of the duration.

### 4.4. Intrusion Trace Efforts under Frequency Conditions

The intrusion trace consists of a series of mutually independent elementary intrusions in a time window which have the Markov property [50]. According to the addition principle in combinatorics, the intrusion trace effort *Effort* is proposed as:

(13) $$Effort=\sum_{i=1}^{rate}ElemEffort$$

where *Effort* represents the intrusion trace effort, and *rate* represents the total count of the elementary intrusions, and *ElemEffort* represents the elementary intrusion effort.

The malicious behavior may cause the Mean-Time-Between-Failures (MTBF) to be shorter [54]. The exponential distribution [55] is used to model the time between the occurrence of events in an interval of time, or the distance between events in space. The exponential distribution has the property of being memoryless [56], which is often used to describe the MTBF distribution of large, complex systems. On the assumption that the potential hacker will eventually succeed in obtaining illegal privileges on an intrusion trace and be willing to expend enough effort to do so, the effort *f*(*Effort*) [21] has the nature of a negative exponential distribution described by:

(14) $$f(Effort)=\{\begin{array}{cc} \lambda e^{-\lambda \times Effort}, & Effort>0,\;\lambda >0\;\\ 0, & Effort\leq 0 \end{array}$$

where *Effort* represents the intrusion trace effort, and λ for a negative exponential distribution, which is the success probability assigned to the elementary intrusion, and *e* is the number 2.71828 …, the base of the natural logs. By the cumulative distribution function, the probability ${Pr}_{i}(Effort)$ [21] that the time between events is less than a specified time *Effort* is given as:

(15) $$Pr_{i}(Effort)=1-e^{-\lambda_{i}\times Effort}$$

The mean or expected value *E_i_*(*Effort*) [21] of an exponentially distributed random variable *Effort* with rate parameter *λ_i_* is given as:

(16) Ei(Effort)=1λi

In general, the harder it is for the malicious behavior to happen, the lower the probability of a successful invasion is. The probability *λ_i_* [21] of a successful intrusion can be represented by the inverse of the degree of difficulty *d_i_* as:

(17) $$\lambda_{i}=\frac{1}{1/d_{i}}=d_{i}\;,d_{i}\in [0,1]$$

The degree of difficulty for launching an elementary intrusion is divided into 10 levels as listed in Table 4 [21].

### 4.5. The Algorithms of Intrusion Effort

**Figure 2:**
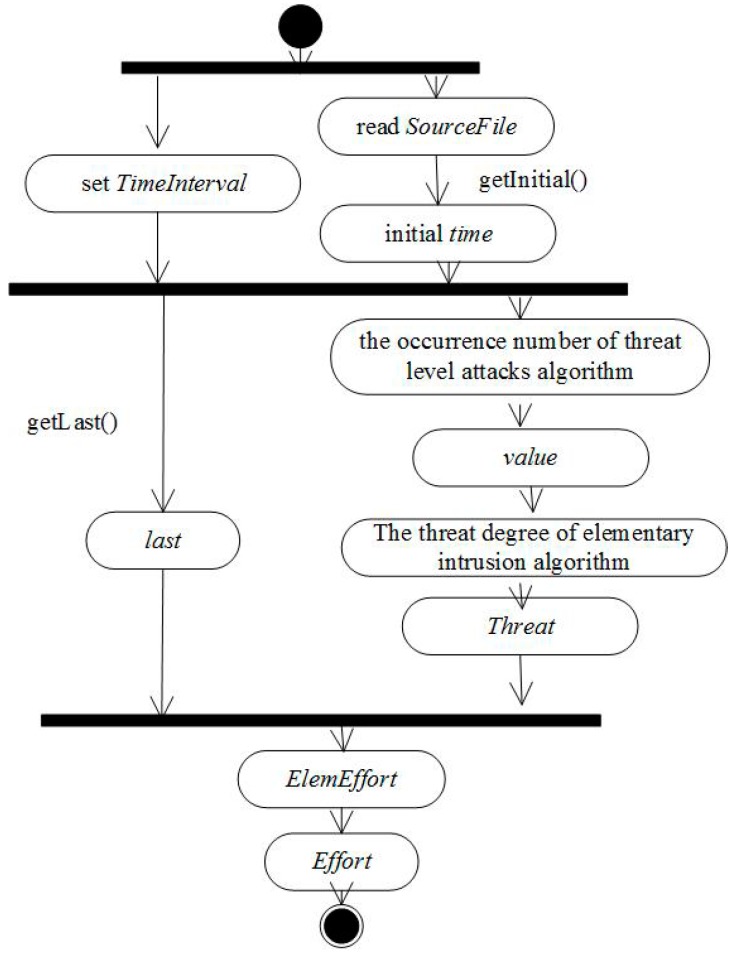
Intrusion effort activity diagram.

The activity diagram of intrusion effort is shown as Figure 2.

Step 1 The time window is set, and the duration of elementary intrusions is obtained one by one according to the final data file in the Handel Data.
Step 2 The harm degree of a malicious act is graded based on Snort user manual and quantified in equidistant divisions.
Step 3 The elementary intrusion effort under threat degree is quantified based on network security situation assessment model, in which the influence coefficient of risk indexes is amended.
Step 4 Based on the elementary intrusion effort, the intrusion trace effort under frequency is quantified based on multiple behavior information fusion.

The algorithm of intrusion effort can be described as follows (Algorithm 1):

**Algorithm 1:** Intrusion trace effort algorithm**Input:**
*TimeInterval*, *SourceFilePath*, *TargetFilePath*. **Output:**
*TargetFileData*. 1. Read *SourceFile* using the class of BufferedReader; 2. Assign by *SourceFile* to the variables of *Begin*, *Last*, and *Degree*, whose types are respectively Stack < Integer >, Stack < Double >, Stack < Float >; 3. Set *TimeInterval* by expert and assign to the variable of *Interval*; 4. The time is initialized as follows: 5. public int getInitial(){ 6. int *time* = begin.get(0); 7. int *result* = *time*/5; 8. int *initial* = result*5; 9. return *initial*;} 10. The elementary intrusion effort is calculated as follows: 11. int *time* = getInitial(); 12. int *ptime* = *time* + (getInterval() − 1); 13. for (int *j* = 0; *j* < begin.size(); *j*++){ 14. if ((begin.get(*j*) <= *ptime*&&begin.get(j) > *time*)||(begin.get(j) > (*time* + 60)&&begin.get(j) < 60)){ 15. *rate*++; 16. The algorithm to calculate the occurrence number of different threat level attacks; 17. The algorithm to calculate the threat degree of elementary intrusion; 18. The algorithm to calculate the elementary intrusion effort integrating last and threat degree; 19. *e* += *ElemEffort*;} 20. else { 21. if (*rate* ! = 0){ 22. Calculate *tracep*;} 23. else { 24. *tracep* = 0;} 25. Store the values of *tracep* and *rate* on the stack; 26. Based on the time windows, the elementary intrusions is divided into the intrusion trace. The intrusion trace effort is calculated as follow: 27. Write into the file of *TargetFile*; 28. Initialization the parameters of *rate*, *e*, *unknown*, *lowest*, *low*, *medium*, *high* is 0; 29. *time* = *ptime*; 30. *ptime* += getInterval(); 31. if (*ptime* > 59) { 32. *time* = *time* - 60; 33. *ptime* = *ptime* - 60;} 34. *j* =*j* - 1;}}

The algorithm to the occurrence number of threat level attacks based on Table 3 can be described as follows (Algorithm 2):

**Algorithm 2:** The occurrence number of threat level attacks algorithm**Input:**
*ThreatDegree*. **Output:** The Stack of Integer *value*. 1. if the input value is 0.2, then{ 2. *unknown*++; 3. push (*unknown*);} 4. else if the input value is 0.4, then{ 5. *verylow*++; 6. push (*verylow*++);} 7. else if the input value is 0.6, then{ 8. *low*++; 9. push (*low*);} 10. else if the input value is 0.8, then{ 11. *medium*++; 12. push (*medium*);} 13. else if the input value is 1.0, then{ 14. *high*++; 15. push(*high*);} 16. else printf (“the value is illegal”); 17. reuturn *value*.

The algorithm to the threat degree of elementary intrusion based on Equation (10) can be described as follows (Algorithm 3):

**Algorithm 3:** The threat degree of elementary intrusion algorithm**Input:** The Stack of Integer *value*. **Output:**
*threat*. 1. The number of different priorities attack from different source at the same time is stored in the object value; 2. *h* = *value*.get(4); 3. *m* = *value*.get(3); 4. *l* = *value*.get(2); 5. *lst* = *value*.get(1); 6. *k* = *value*.get(0); 7. for (int *i* = 0; *i* < 5; *i*++){ 8. *sum_1_* = h*Math.pow(10, 5*1) + m*Math.pow(10, 4*0.8) + l*Math.pow(10, 3*0.6) + lst*Math.pow(10, 2*0.4) + k*Math.pow(10, 1*0.2); 9. int *sum* = *h* + *m* + *l* + *lst* +*k*; 10. if(*sum*==0){ 11. return 0;}} 12. double *weight* = *sum*_1_/(sum*100); 13. double *threat* = Math.pow(10, *weight*); 14. Return *threat*.

The algorithm of the elementary intrusion effort integrating last and threat degree based on Equation (12) can be described as follows (Algorithm 4):

**Algorithm 4:** The elementary intrusion effort integrating last and threat degree algorithm**Input:**
*TimeLast*, *ThreatDegree*. **Output:**
*ElemEffort*. 1. The dimensionless of attack duration *last* is treated: 2. *last* = *lasttime*/Math.pow(10, *j*); 3. *ElemEffort* = *threat* + *last;* 4. Return *ElemEffort*.

## 5. A Quantitative Risk Assessment Model Involving Frequency and Threat Degree

Deployed on a SP’s server over the Internet, services can be accessed by users. Each access event represents a user behavior, which affects LoBSs’ risk. The user behaviors of different frequency and threat degree impact on LoBSs differently. An elementary intrusion is any alert that is evoked by intrusion behavior and reported by an intrusion detection system. An intrusion trace is a trace constituted of a series of elementary intrusions that user privilege has led to getting illegally. On the basis of intrusion trace effort, QRAM involving frequency and threat degree under LoBSs is proposed based on VaR.

### 5.1. Line-of-Business Services’ Risk involving Frequency and Threat Degree

LoBSs’ risk is impacted by many factors, so it has many evaluation methods. Its main evaluation methods include the subjective risk evaluation method based on subjective data supplied by experts’ scoring and the objective risk evaluation method based on the data detected while running LoBSs. Integrating the advantages of both a subjective risk evaluation method and an objective risk evaluation method, a comprehensive risk evaluation method is proposed which is more suitable for LoBSs.

#### 5.1.1. An Objective Risk Evaluation Method

The occurrence of malicious behavior is often driven by interest. The rate of weighted threat in intrusion traces has different effects on LoBSs’ risk. In general, the greater is the rate of a weighted threat in an intrusion trace, the greater is its effect on LoBSs’ risk.

The time window frequency (Definition 5) under different threat degree is defined as:

(18) $$\{\begin{array}{c} \begin{array}{c} highp=high/rate\\ mediump=medium/rate \end{array}\\ lowp=low/rate\\ \begin{array}{c} verylowp=verylow/rate\\ unknownp=unknown/rate \end{array} \end{array}$$

where *highp*, *mediump*, *lowp*, *verylowp* separately represent the frequency of threat degree in an intrusion trace, and *high*, *medium*, *low*, *verylow*, *unknown* separately represent the generation numbers of threat degree in an intrusion trace, and *rate* represents the total generation number of elementary intrusions in an intrusion trace.

Based on Table 3 and Equation (18), the rate of weighted threat in an intrusion trace *Tracep* (Definition 6) is defined as:

(19) Tracep=highp×1+mediump×0.8+lowp×0.6+verylowp×0.4+unknownp×0.2

Corresponding to the degree of difficulty of an elementary intrusion divided into 10 levels, the objective risk *I_o_* was quantified in 1, 2, ..., 10 by the equidistant division of *Tracep* following as:

(1) The objective risk *I_o_* = 10, if *Tracep* ≥ 90%;
(2) The objective risk *I_o_* = 9, if 90% > *Tracep* ≥ 80%;
(3) The objective risk *I_o_* = 8, if 80% > *Tracep* ≥ 70%;
(4) The objective risk *I_o_* = 7, if 70% > *Tracep* ≥ 60%;
(5) The objective risk *I_o_* = 6, if 60% > *Tracep* ≥ 50%;
(6) The objective risk *I_o_* = 5, if 50% > *Tracep* ≥ 40%;
(7) The objective risk *I_o_* = 4, if 40% > *Tracep* ≥ 30%;
(8) The objective risk *I_o_* = 3, if 30% > *Tracep* ≥ 20%;
(9) The objective risk *I_o_* = 2, if 20% > *Tracep* ≥ 10%;
(10) The objective risk *I_o_* = 1, if 10% > *Tracep* ≥ 0.

The integer interval of objective risk is [1, 10]. The objective risk *I_o_* can be given by the rate of weighted threats in an intrusion trace. For example, suppose that the rate of a weighted threat in a *j*-th intrusion trace *Tracp*_j_ is 0.75, then the objective risk *I_oj_* is 8.

#### 5.1.2. A Subjective Risk Evaluation Method

A subjective risk evaluation is calculated by experts’ scores. In order to ensure consistency with the objective risk, the interval of expert’s score limits is [1, 10]. Supposing that expert’s score set is *U* = {1, 2, 3, 4, 5, 6, 7, 8, 9, 10}, and there are *m* experts to score *n* elemental intrusions of an intrusion trace, then the expert score matrix for this intrusion trace is described as:

(20) $$score=\left[\begin{array}{ccc} score_{11} & \cdots & score_{1n}\\ \vdots & \ddots & \vdots \\ score_{m1} & \cdots & score_{mn} \end{array}\right]$$

where *score_ij_* represents the score of the *i*-th expert for the *j*-th element intrusion of an intrusion trace, $1\leq i\leq m,\;1\leq j\leq n,\;1\leq score_{ij}\leq 10$.

It can be seen from Equation (20) that the score set of expert *m* for the *j*-th element intrusion of an intrusion trace is *score_j_* = {*score*_1*j*_, *score*_2*j*_, …, *score_mj_*}. The expert’s score *S_j_* of the *j*-th element intrusion of an intrusion trace is averaged by expert *m*’s score as:

(21) $$S_{j}=\frac{score_{1j}+socre_{2j}+\ldots +score_{mj}}{m}$$

Since the interval of *score_ij_* (1≤i≤m, 1≤j≤n) is between [1, 10], it is normalized as:

(22) $$p_{ij}=\frac{score_{ij}}{score_{1j}+score_{2j}+score_{mj}},\;1\leq i\leq m,\;1\leq j\leq n,\;1\leq score_{ij}\leq 10$$

The expert scoring matrix for intrusion trace is transformed as:

(23) $$score^{\prime }=\left[\begin{array}{ccc} p_{11} & \cdots & p_{1n}\\ \vdots & \ddots & \vdots \\ p_{m1} & \cdots & p_{mn} \end{array}\right],\;p_{1j}+p_{2j}+\ldots +p_{mj}=1,\;j=1,2,..,n$$

Since an intrusion trace comprises a lot of elementary intrusions which are evaluated by experts, the subjective risk based on Shannon entropy [37] of expert score matrix *H*_j_ is proposed as:

(24) $$H_{j}=-\frac{1}{\ln m}\sum_{i=1}^{m}p_{ij}\ln p_{ij}\;,\;j=1,2,\ldots ,n$$

The subjective risk *I_s__j_* of the *j*-th intrusion trace is calculated as:

(25) $$I_{sj}=H_{j}\times S_{j}$$

#### 5.1.3. A Comprehensive Risk Evaluation Method

Objective risk evaluation methods are susceptible to the bias of sample data. Subjective risk evaluation methods are susceptible to experts’ subjectivity. Integrating the advantages of subjective risk and objective risk, a comprehensive risk evaluation method is proposed, based on the intrusion trace probability and the proportion between subjective risk and objective risk which is evaluated by experts. If the interval of subjective risk *p*_1*j*_, *p*_2*j*_, …, *p_mj_* undulates violently, it is shown that expert’s evaluations are serious differences. The effectiveness of subjective risk is weak, and the proportion of subjective risk in the comprehensive risk should be reduced. On the contrary, the proportion should be increased.

Supposing that the subjective risk’s weight of the *j*-th intrusion trace in the comprehensive risk is *W_sj_*, the weight of objective risk *W_oj_* is calculated as:

*W_oj_* = 1 − *W_sj_* (26)

The comprehensive risk *I*_c*j*_ of the *j*-th intrusion trace is calculated as:

(27) $$I_{cj}=W_{sj}\times I_{sj}+W_{oj}\times I_{oj}$$

The comprehensive risk is limited to [1, 10], which is normalized as:

(28) $$r_{j}=I_{cj}/10$$

The change rate *Q_j_* of risk affecting function is defined by the probability *Pr_j_* (Equation (15)) of intrusion trace multiplied by the normalization *r_j_* of comprehensive risk, that is:

(29) $$Q_{j}={Pr}_{j}\times r_{j}$$

### 5.2. A Quantitative Risk Assessment Model

Just like financial assets or a portfolio may lose value due to market fluctuations, users’ behavior may lead to LoBSs’ risk. Based on VaR [18] which is commonly used in financial risk assessment, QRAM involving frequency and threat degree is proposed to quantify LoBSs’ risk.

The keys to calculating VaR include the forecast of future market changes and the relationship between the portfolio and the market (linearity, non-linearity). The fundamental calculation methods of forecasting future market changes include historical simulation method, parametric method and the Monte Carlo method [45], whose advantages can be listed as follows:

The implicit assumptions of parametric method are a normal distribution and the invariance of volatility and correlation, but when the number of assets in the portfolio is large, it is difficult to ensure that variance and covariance [45].

Based on stochastic simulations, the Monte Carlo method has many shortcomings, such as the choice of models, the quality of random numbers, relying on a particular stochastic process, etc. [45].

The core of historical simulation method is to simulate the future income distribution of the portfolio based on the historical sample changes, and then uses the quartile to calculate the VaR under a certain degree of confidence [45]. Historical simulation method calculates the total value of the portfolio, rather than the local approximation of small changes in price. At the same time, the historical simulation method avoids the simulation risk by using real data, and it do not need to make specific assumptions on the distribution, nor do it need to estimate the parameters, so it can deal with asymmetric and fat tail problems. In addition, as the historical data reflects the simultaneous changes of all risk factors in the market, the problems of volatility, relevance, and fat tail can be reflected by the data.

Based on the comprehensive analysis of the three methods, in this work LoBSs’ risk is quantified by the historical simulation method of VaR. Supposing that the initial risk of LoBSs is *R*_0_, and the change rate of the risk affecting function is *Q_j_* (Equation (29)), the risk *R* after happening the *j*-th intrusion trace is calculated as:

*R* = *R*_0_ (1 − *Q_j_*) (30)

Supposing that the confidence level (Definition 8) is *c*, and the highest risk is R^*^ = *R*_0_ (1 − Q_j_^*^), and the expectation of *Q_j_* (Equation (29)) is *u_j_*, and the expectation of *R* is *E*(*R*), then LoBSs’ VaR *VaR*_R_ based on Equation (4) is proposed as:

(31) $$VaR_{R}=E(R)-R^{*}=R_{0}(1-u_{j})-R_{0}(1-Q^{*})=R_{0}(Q^{*}-u_{j})$$

In the other words, to calculate VaR is equivalent to calculate the maximum risk R^*^ or the minimum change rate of risk affecting function Q^*^ of LoBSs. The probability density function *f*(*R*) of LoBSs’ risk change can be calculated based on *R* = *R*_0_ (1 − *Q_j_*). Based on Equation (6), the maximum risk R^*^ for LoBSs at a certain confidence level *c* (Definition 8) is defined as:

(32) $$c=\int_{0}^{R^{*}}f(R)dR$$

### 5.3. The Algorithm to Assess Line-of-Business Services’ Risk

**Figure 3:**
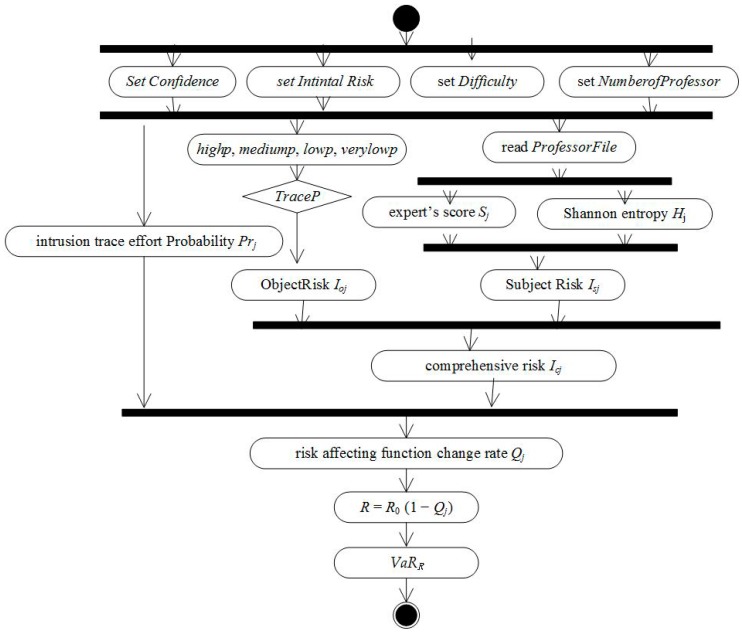
Assessing LoBSs’ risk activity diagram.

The activity diagram of LoBSs’ risk assessment is shown in Figure 3.

Step 1 The parameters of confidence degree, initial risk, operational difficult degree, number of expert are initialized.
Step 2 The objective risk are calculated according to the intrusion trace effort.
Step 3 The subjective risk was calculated according to the Shannon entropy of experts’ scores.
Step 4 A comprehensive risk is combined with objective risk and subjective risk.
Step 5 The rate of risk impact is calculated by combining the comprehensive risk with the probability of intrusion trace.
Step 6 LoBSs’ risk is calculated by the historical simulation method of VaR.

The algorithm to assess LoBSs’ risk can be described as follows (Algorithm 5):

**Algorithm 5:** LoBSs’ risk assessment algorithm**Input:**
*SourceFilePath*, *ProfessorFilePath*, *WeightFilePath*, *InitialRisk*, *Confidence*, *Difficulty*, *NumberofProfessor*. **Output:**
*VaR_R_*. 1. Assign the parameters of *InitialRisk*, *Confidence*, *Difficulty*, *NumberofProfessor*; 2. Read *SourceFile* Using BufferedReader; 3. the ObjectiveRisk *I_oj_* is get by judging *TraceP*,then, push into the corresponding stack; 4. Read *ProfessorFile* using BufferedReader; 5. The files in *ProfessorFile* are stored with the type of List <double []>, the attack Shannon entropy is calculated; 6. Calculating the subjective risk *I_sj_*; 7. Read *WeightFile* using BufferedReader, the comprehensive risk *I_cj_* is calculated based on the weight between subjective risk and objective risk; 8. The rate of risk impact is calculated by $Q_{j}={Pr}_{j}\times r_{j}$; 9. *VaR_R_* is calculated based on VaR; 10. Return *VaR_R_*.

## 6. Simulation Test and Discussion

In order to test QRAM, a prototype is designed based on the unified modeling language, and implemented based on Java. In order to verify QRAM, we would need some data from a SP’s server, but we cannot get. In SP server of LoBSs, the services are centrally provided to users by the multi-tenant model which are used by tenant’s users over the Internet, so users are out of tenant entity management domain when using a SP’s service. The behavior characteristics of user are similar to those in a traditional network system, so for a test it is suitable to simulate LoBSs using a traditional information system, whose data comes from the simple data of Windows NT attack data set (Sim-Data-NT) in 2000 defense advanced research projects agency intrusion detection evaluation data set of massachusetts institute of technology lincoln laboratory [57].

### 6.1. Simulation Data

There are 358 elementary intrusion data in Sim-Data-NT [57], whose threat level includes six high, 46 medium, 306 unknown, and hardly any low and very low. Because there is hardly any data of threat level both low and very low, it is not consistent with reality. In order to be consistent with the real situation, the set of Sim-Data-NT should be optimized. Supposing that SMTP packets are altered by an icmp-event attack, and the FTP packets are altered by a tcp-connetion attack, according to Snort default classification [19], there are 358 elementary intrusion data in the optimized Sim-Data-NT, whose threat level includes six high, 46 medium, 24 low, 18 very low, 264 unknown. According to Table 3, the threat degree of test data in the optimized Sim-Data-NT is quantified.

Because the information of the optimized Sim-Data-NT is imperfect as it cannot determine the intrusion trace constituted by the elementary intrusions, it is assumed that the elementary intrusions within the time window constitute an intrusion trace. By testing the intrusion trace effort for different time windows (such as 1 s, 10 s, 20 s, etc.), the suitable maximum and minimum time windows can be obtained. An intrusion trace is simulated by randomly splitting the time windows between the maximum and the minimum.

**Figure 4:**
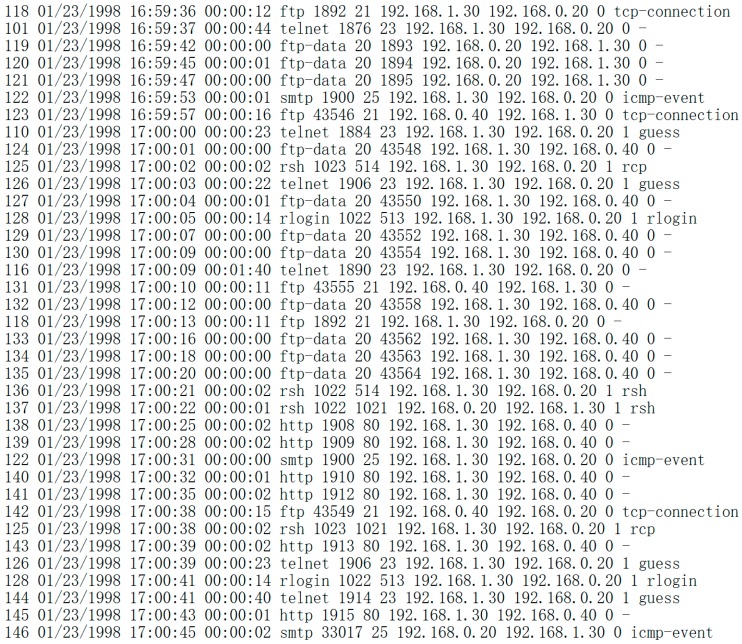
Part of source data.

**Figure 5:**
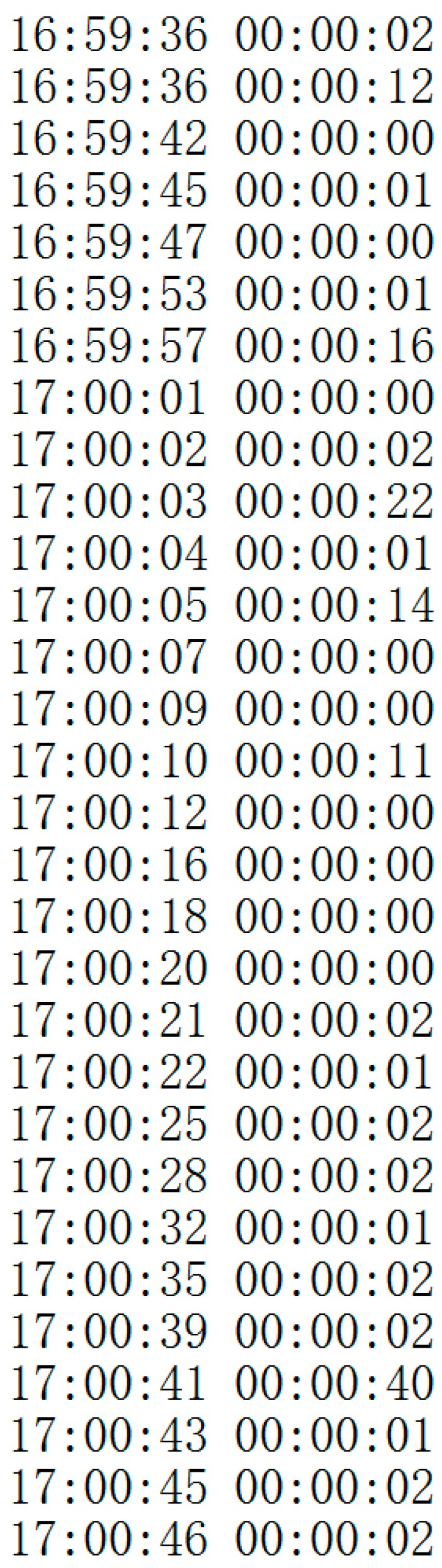
Part of source data including attack time, duration and type.

In order to facilitate post-processing, the ternary coding system is adopted, for example the number 1 is coded as 001. The source data including the parameters of attack time, duration and type are selected as seen in Figure 4 and Figure 5. By the Snort default classification [19], the threat degree of attack is quantified as shown in Table 5.

### 6.2. Testing and Results

**Figure 6:**
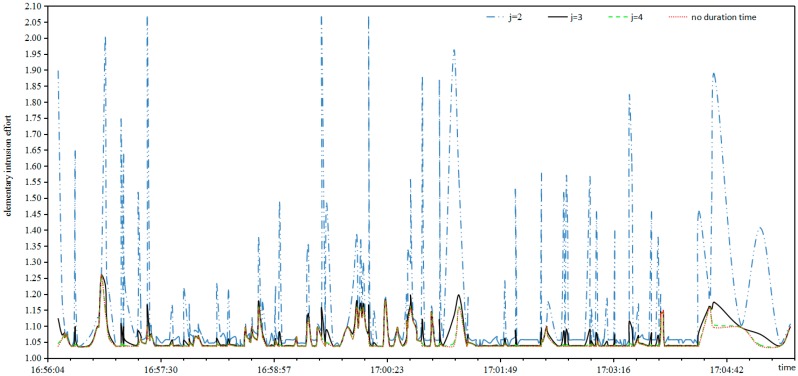
Relationship between elementary intrusion effort and duration.

Based on the optimized Sim-Data-NT, the simulation test items by our prototype system include: (1) elementary intrusion effort; (2) intrusion trace effort; (3) objective risk; (4) subjective risk; (5) comprehensive risk; (6) LoBSs’ Quantitative Risk. The simulation testing and results are as follows:

- *Elementary* *Intrusion* *Effort*: Suppose the parameter *j* of Equation (11) is respectively assigned values of 2, 3, 4, then the relationship between elementary intrusion effort and duration based on Equation (12) is as shown in Figure 6.

It can be seen from Figure 6 that:

- The cures of elementary intrusion effort deviate greatly between *j* = 2 and no duration, that is, the duration interferes with the elementary intrusion effort too much.
- The cures of elementary intrusion effort hardly coincide between *j* = 4 and no duration, that is, the duration interferes with the elementary intrusion effort next to nothing.
- The cures of elementary intrusion effort are almost synchronized between *j* = 3 and no duration, that is, the duration strengthens the elementary intrusion effort.

It can be concluded that *j* = 3 is suitable for the experiment because it takes into account the effects of both threat degree and duration, that is, the duration influence on the elementary intrusion effort is reasonable when the normalized parameter is 1000.

**Figure 7:**
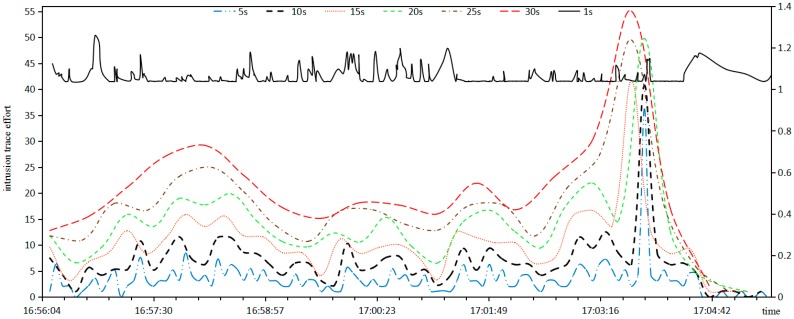
Relationship between intrusion trace effort and time widow.

- *Intrusion* *Trace* *Effort*: Suppose that the time window is respectively assigned as 1 s, 5 s, 10 s, 15 s, 20 s, 25 s, 30 s, the relationship between intrusion trace effort and time widow of Equation (13) is shown in Figure 7.

In Figure 7, the abscissa axis represents the time, the principal left ordinate represents the invasion trace effort of unit 5; The auxiliary right ordinate represents the invasion trace effort of unit 0.2; Except that the time window 1 s lies in the auxiliary right ordinate, the others lie in the principal left ordinate.

It can be seen from Figure 7 that:

- When the time window is 1 s, an intrusion trace only includes an elementary intrusion, that is, an intrusion trace degenerates to an elementary intrusion, and the intrusion trace effort fluctuates with high-frequency.
- When the time window is 5 s, like an elementary intrusion, the intrusion trace effort fluctuates with high-frequency.
- When the time window is 30 s, the curve of intrusion trace effort is level and smooth, and many malicious attacks are smoothed and therefore skipped.
- When the time window is 10 s, the tendency of the intrusion trace effort coincides with the elementary intrusion effort.

It can be concluded that 10 s are suitable for the time window of intrusion traces which can effectively avoid the curve fluctuations in small time window, but avoid the curve smoothing in a large time window.

**Figure 8:**
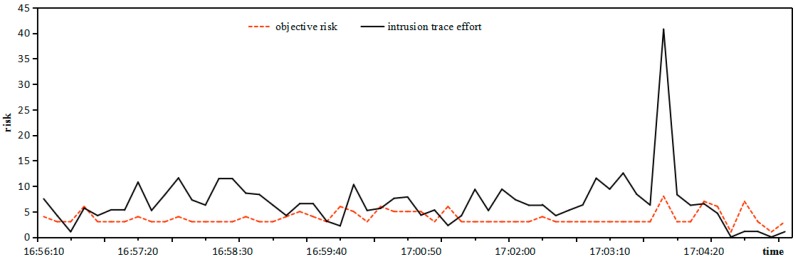
Relationship between intrusion trace effort and objective risk.

- *Objective* *Risk***:** The objective risk is calculated by the rate of weighted threat in an intrusion trace, and the relationship between intrusion trace effort and objective risk is shown in Figure 8.

It can be seen from Figure 8 that the tendencies between intrusion trace effort and objective risk coincide in the overwhelming majority of cases. Only in specific individual time intervals the objective risk is inactivated since the intrusion trace effort fluctuates little, so it is practical to estimate the objective risk by the rate of weighted threat in the intrusion trace.

**Figure 9:**
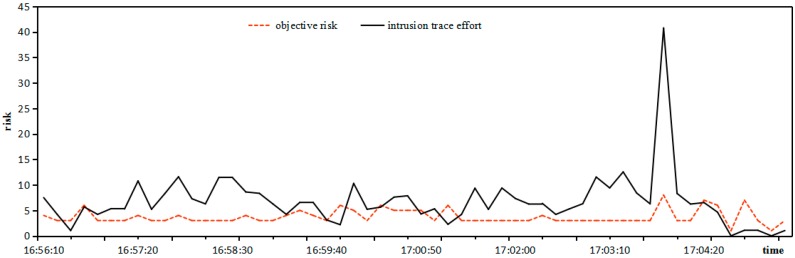
Relationship between intrusion trace effort and subjective risk.

- *Subjective* *Risk*: The subjective risk is calculated by Shannon entropy based on the experts’ scoring matrix, then the relationship between intrusion trace effort and subjective risk is shown in Figure 9.

It can be seen from Figure 9 that the tendency between intrusion trace effort and subjective risk coincides in the overwhelming majority of cases. Only in specific individual time intervals the subjective risk is inactivated since the intrusion trace effort fluctuates little, so it is practical to estimate the subjective risk by Shannon entropy based on the experts’ scoring matrix.

**Figure 10:**
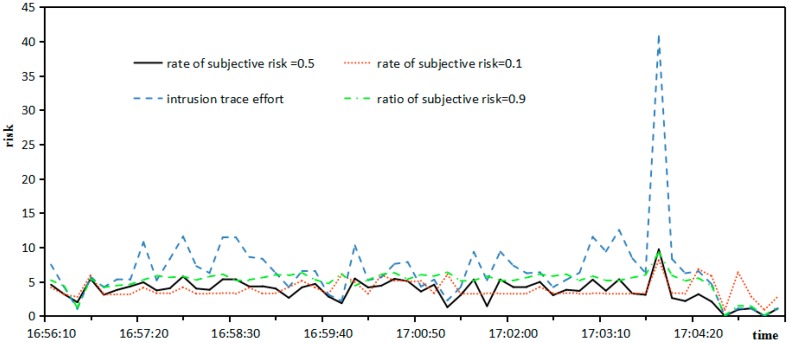
Comprehensive risk under different ratios between objective risk and subjective risk.

- *Comprehensive* *Risk*: The comprehensive risks under different ratio between objective risk and subjective risk are shown as Figure 10.

It can be seen from Figure 10 that when the ratio of objective risk is 0.5, the tendency between intrusion trace effort and comprehensive risk coincides, so it is practical to adopt a comprehensive risk between objective risk and subjective risk under a ratio of 0.5.

**Figure 11:**
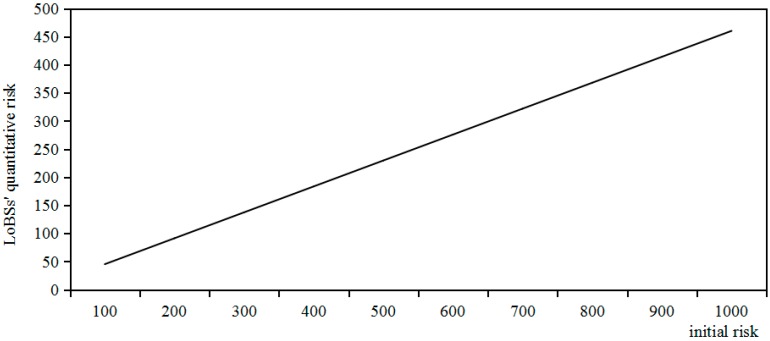
Relationship between initial risk and LoBSs’ quantitative risk.

- *LoBSs’* *Quantitative* *Risk*: Under the conditions of confidence level, number of experts, attack difficulty degree, initial risk, and time window, the change tendency of LoBSs’ quantitative risk based on QRAM is individually investigated.
- Under the condition that the confidence level is 95%, and the number of experts is 5, and the attack difficult degree is 0.1, the relationship between initial risk and LoBSs’ quantitative risk based on QRAM is shown in Figure 11.

It can be seen from Figure 11 that there is a linearly-increasing relation between initial risk and LoBSs’ quantitative risk based on QRAM.

**Figure 12:**
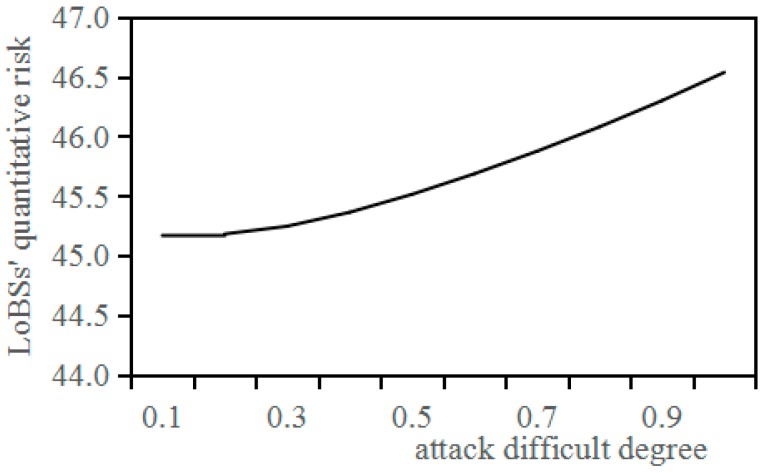
Relationship between attack difficulty degree and LoBSs’ quantitative risk.

- Under the condition that the confidence level is 95%, and the number of experts is 5, and the initial risk is 100, the relationship between attack difficult degree and LoBSs’ quantitative risk based on QRAM is shown in Figure 12.

It can be seen from Figure 12 that LoBSs’ quantitative risk based on QRAM hardly changes when the attack difficulty degree is less than 0.3, and it can be quantified in accordance with the quadratic polynomial *y* = 0.0127*x*^2^ + 0.018*x* + 45.108.

**Figure 13:**
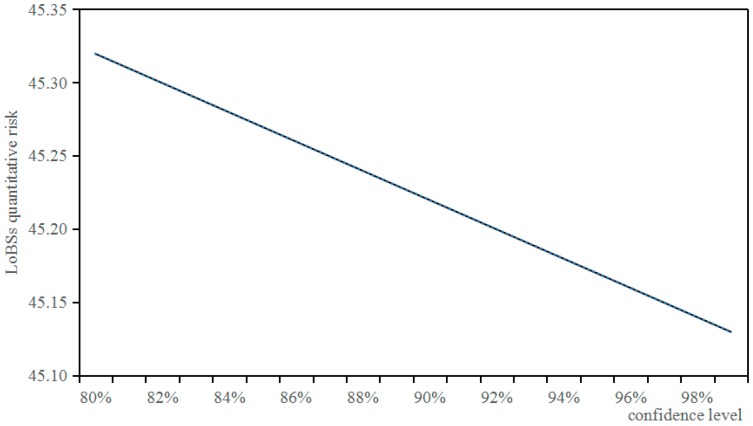
Relationship between confidence level and LoBSs’ quantitative risk.

- Under the condition that the number of experts is 5, and the initial risk is 100, and the attack difficulty degree is 0.1, the relationship between confidence level and LoBSs’ quantitative risk based on QRAM is shown in Figure 13.

It can be seen from Figure 13 that there is a linearly-decreasing relation between confidence level and LoBSs’ quantitative risk based on QRAM. The lower the confidence level is, the rougher LoBSs’ quantitative risk is; the higher the confidence level is, the more accurate LoBSs’ quantitative risk is.

**Figure 14:**
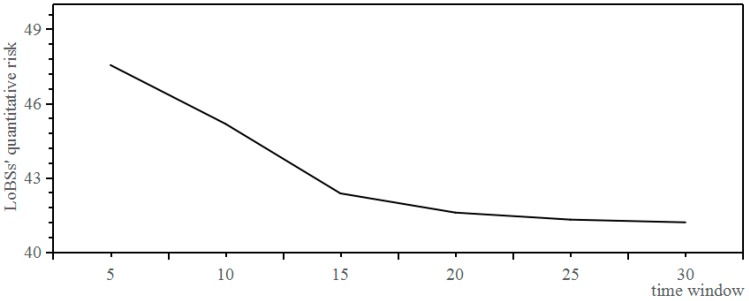
Relationship between time window and LoBSs’ quantitative risk.

- Under the condition that the number of experts is 5, and the initial risk is 100, and the attack difficulty degree is 0.1, and the confidence level is 95%, the relationship between time window and LoBSs’ quantitative risk based on QRAM is shown in Figure 14.

It can be shown from Figure 14 that LoBSs’ quantitative risk based on QRAM hardly changes when the time widow is larger than 15 s, and it can be quantified in accordance with the quadratic polynomial *y* = −0.0146*x*^3^ + 0.5356*x*^2^ − 4.4012*x* + 51.543. The study just selects the data of elementary intrusions rather than the intrusion trace in the optimized Sim-Data-NT. The intrusion traces are defined by the elementary intrusions in a splitting time window. The splitting time window will influence the constitution of the intrusion traces, and furthermore its effort, but hardly affect LoBSs’ quantitative risk based on QRAM.

## 7. Conclusions

As one of cloud computing’s service models, SaaS is one of development directions of software delivery. As one of SaaS’s service types, LoBSs are often large, customizable business solutions offered to enterprises and organizations and aimed at facilitating business processes. A lot of valuable resources are accumulated on SP’s server, so the access permission is a top priority in LoBSs’ security, which is one of the biggest challenges to LoBSs. LoBSs’ users are very diverse as they may come from a wide range of locations with vastly different characteristics. The cost of joining could be low and in many cases, the intruders are just eligible users conducting malicious actions. In order to dynamically adjust user access, LoBSs’ risk must be dynamically assessed. Both frequency and threat degree of malicious operation have an important effect on LoBSs’ risk. The higher is the frequency of malicious operations, the higher is the risk of LoBSs. The larger is the threat degree of malicious actions, the greater is the risk of LoBSs. In order to quantify LoBSs’ risk more precisely, the impact both frequency and threat degree of user behavior must be considered.

Based on VaR, QRAM involving frequency and threat degree is proposed under LoBSs for infrastructure of ESNs. The degree of harm of a malicious act is graded based on Snort user manual [19] and quantified in equidistant divisions. The elementary intrusion effort under threat degree is quantified based on a network security situation assessment model [20], in which the influence coefficient of risk indexes is amended. The intrusion trace effort under frequency is quantified based on multiple behavior information fusion [21]. The objective risk of LoBSs is quantified based on the rate of weighted threat in intrusion traces. The subjective risk of LoBSs is quantified based on Shannon entropy [38] of experts’ scores. The comprehensive risk of LoBSs is quantified on both the intrusion trace probability and the proportion between subjective risk and objective risk. Under the influence of intrusion trace, LoBSs’ risk is accessed dynamically by the historical simulation method of VaR.

In order to perform a simulation test, a prototype is designed based on the unified modeling language, and implemented based on Java. Based on the optimized Sim-Data-NT, simulation testing by the prototype, it can be shown that the duration influence on elementary intrusion effort is reasonable when the normalized parameter is 1000; 10 s are suitable for the time window of intrusion trace; the comprehensive risk can be correctly reflected when the weight ratio between objective risk and subjective risk is 0.5. Under the conditions of confidence level, number of expert, attack difficulty degree, initial risk and time window, after the change tendency of LoBSs’ quantitative risk is respectively tested, LoBSs’ risk can be assessed dynamically by QRAM involving frequency and threat degree.

QRAM involving frequency and threat degree focuses on LoBSs for infrastructure of ESNs, and may promote to other cloud computing scenarios, but there are more factors such as financial cost, attacker’s experience, practicability of attack tool, counting ability, and so on, which may influence on LoBSs’ risk, and should be involved in any evaluation.

## Figures and Tables

**Table 1:** Definitions of elements for elementary intrusion, intrusion trace, network packet and network session [21].

Elements	Definitions
*aid*	sequence number of intrusion event
*src/dst*	source/destination address
*sp/dp*	source/destination port
*t*	occurrence time
*type*	event type
*sensor*	name of intrusion detection sensor
*count*	occurrence times of one elementary intrusion in one session
*pid/cid*	sequence number of network packet/intrusion trace
*flag*	TCP sign
*pro*	communication protocol in the transport layer
*load*	content of network packet
*sid*	sequence number of network session
*sig*	signature of intrusion event
*seq*	sequence number of elementary intrusion in one intrusion trace

**Table 2:** Snort default classification [19].

Class Type	Description	Priority
attempted-admin	Attempted Administrator Privilege Gain	high
attempted-user	Attempted User Privilege Gain	high
inappropriate-content	Inappropriate Content was Detected	high
policy-violation	Potential Corporate Privacy Violation	high
shellcode-detect	Executable code was detected	high
successful-admin	Successful Administrator Privileges Gain	high
successful-user	Successful User Privilege	high
trojan-activity	A Network Trojan was detected	high
unsuccessful-user	Unsuccessful User Privilege Grain	high
web-application-attack	Web Application Attack	high
attempted-dos	Attempted Denial of Service	medium
attempted-recon	Attempted Information Leak	medium
bad-unknown	Potentially Bad Traffic	medium
default-login-attempt	Attempt to login by a default username and password	medium
denial-of-service	Detection of a Denial of Service Attack	medium
misc-attack	Misc Attack	medium
non-standard-protocol	Detection of a non-standard protocol or event	medium
rpc-portmap-decode	Decode of an RPC Query	medium
successful-dos	Denial of Service	medium
successful-recon-large-scale	Large Scale Information Leak	medium
successful-recon-limited	Information Leak	medium
suspicious-filename-detect	A suspicious filename was detected	medium
suspicious-login	An attempted login using a suspicious username was detected	medium
system-call-detect	A system call was detected	medium
unusual-client-port-connection	A client was using an unusual port	medium
web-application-activity	Access to a potentially vulnerable web application	medium
icmp-event	Generic ICMP event	low
misc-activity	Misc activity	low
network-scan	Detection of a Network Scan	low
not-suspicious	Not Suspicious Traffic	low
Protocol-command-decode	Generic Protocol Command Decode	low
String-detect	A suspicious string was detected	low
unknown	Unknown Traffic	low
tcp-connection	A TCP connection was detected	very low

**Table 3:** Attack priority quantization.

Priority	Quantization
high	1.0
medium	0.8
low	0.6
very low	0.4
unknown	0.2

**Table 4:** Scale of the degree of difficulty to launch elementary intrusions [21].

Levels	Description	*d_j_*
1	very simple	1
2	relatively simple	0.9
3	fairly simple	0.8
4	simple	0.7
5	non-trivial	0.6
6	not-so trivial	0.5
7	trivial	0.4
8	intermediate	0.3
9	moderate	0.2
10	difficult	0.1

**Table 5:** Threat degree of attack and quantification.

Attack Type	Quantization
-	0.2
phf	1
rsh	1
rcp	1
guess	0.8
rlogin	0.8
port-scan	0.8
portsweep	0.6
icmp-event	0.6
tcp-connection	0.4
